# Supplementary material for: Thalassemys bruntrutana n. sp., a new coastal marine turtle from the Late Jurassic of Porrentruy (Switzerland), and the paleobiogeography of the Thalassemydidae
Source: PeerJ. 2015 Sep 29;3:e1282. doi: 10.7717/peerj.1282 (PMC4592157; doi:10.7717/peerj.1282)
Supplement: Table S1 [file peerj-03-1282-s001.docx]

| **Neurals** | **NJSN SCR011-87** | | **NMS 8595–8609** | |
| --- | --- | --- | --- | --- |
| No. | Median length | Max. width | Median length | Max. width |
| 1 | - | - | 62 | 45 |
| 2 | 70 | 37 | 58 | 44 |
| 3 | 70 | 35 | 67 | 41 |
| 4 | 55 | 35 | 63 | 34 |
| 5 | 53 | 38 | 62 | 36 |
| 6 | 57 | 43 | 61^a^ | 36 |
| 7 | 35 | 41 | 25 | 40 |
| 8 | 29 | 50 | 25 | 40 |

| **Costals** | **NJSN SCR011-87 ^b^** | | **NMS 8595–8609 ^c^** | |
| --- | --- | --- | --- | --- |
| No. | Lateral length | Max. width | Lateral length | Max. width |
| 1 | - | 184 | - | 175 |
| 2 | 105 | 221 | 93 | 235 |
| 3 | 97 | 249 | 93 | 253 |
| 4 | 72 | 252 | 75 | 253 |
| 5 | 89 | 228 | 72 | 230 |
| 6 | - | 168 | 60 | 200 |
| 7 | - | - | - | - |
| 8 | - | - | - | - |

^a^ neural 6 of NMS 8595–8609 is divided into two bones

^b^ left costals

**^c^** right costals (measurements taken from Bräm, 1965)
